# Patterns and Correlates of Sedentary Behavior in Children Attending Family Child Care

**DOI:** 10.3390/ijerph17020549

**Published:** 2020-01-15

**Authors:** Li Kheng Chai, Kelly Rice-McNeil, Stewart G. Trost

**Affiliations:** 1 Institute of Health and Biomedical Innovation (IHBI) at Queensland Centre for Children’s Health Research, Queensland University of Technology, South Brisbane, QLD 4101, Australia; likheng.chai@qut.edu.au; 2 Children’s Health Queensland Hospital and Health Service, South Brisbane, QLD 4101, Australia; 3 Department of Health and Human Performance, Eastern Oregon University, La Grande, OR 97850, USA; krice@eou.edu

**Keywords:** early childhood education and care, sedentary behavior, family child care home, screen time

## Abstract

Public health authorities recommend young children should not be sedentary for more than one hour at a time. This study assessed the frequency and duration of sedentary bouts in children attending family child care homes (FCCHs); and examined associations with FCCH provider practices related to sedentary behaviors. Overall, 127 children (aged 3.5 ± 1.1 years) from 41 FCCHs participated in the study. Sedentary bouts were measured using an accelerometer worn for the duration of FCCHs attendance over a randomly selected week. Provider practices were assessed using the Nutrition and Physical Activity Self-Assessment for Child Care self-assessment instrument. Children attending FCCHs mostly accumulated short sedentary bouts (<5 min) with very few lasting more than 10 min. Boys exhibited significantly fewer sedentary bouts, and significantly less sedentary time in bouts than girls. Children attending FCCHs that met or exceeded childcare standards for outdoor active play, had portable play equipment, offered a variety of fixed play equipment, and/or adequate indoor play space exhibited significantly fewer sedentary bouts and significantly less sedentary time accumulated in short and medium length bouts. Programs encouraging FCCHs to adopt physical activity promoting practices could potentially reduce child sedentary time while in care.

## 1. Introduction

Participation in physical activity during the preschool years is critical for healthy growth and development [1]. Evidence demonstrates that physical activity in children aged five years and under is beneficial for healthy weight, cardio-metabolic health, skeletal health, and cognitive skills development [1]. Regular physical activity in children also helps in promoting healthy weight [2], developing fundamental motor skills, enhancing muscular strength, and learning social skills [3]. In addition, it is increasingly recognized that too much sedentary time can have negative effects on health and a number of countries, including the United States, Canada, Australia, and New Zealand, have national recommendations to limit sedentary behavior, especially screen time, in young children [4].

The World Health Organization recommends children aged 1–5 years should not be restrained (i.e., high chair, stroller) for more than one hour at a time, and children aged 1–4 years should not sit for extended periods of time [4]. However, there is evidence that sedentary behavior is highly prevalent in young children. A longitudinal study in Canada reported that children in kindergarten spent approximately six hours per day (60% of the day) being sedentary [5]. The study also found that time in sedentary behavior increased from kindergarten to grade two [5]. An analysis of the 2003–2004 U.S. National Health and Nutrition Examination Survey (NHANES) accelerometer data indicated that children aged 6–11 years spent approximately six hours per day being sedentary [6]. Similar findings were reported in a cross-sectional study using data from NHANES, which found that physical activity declines dramatically between childhood and adolescence and continues to decline with age [7]. As the development of a physically active lifestyle starts in early childhood and tracks into youth and adulthood [8], a better understanding of the patterns and correlates of physical activity and sedentary behavior in young children is warranted.

In 2011, 61% of U.S. children under the age of five years were in some form of child care arrangement on a regular basis [9]. Although center-based childcare is the most common choice for nonrelative care [9], family child care homes (FCCHs)—defined as a setting where a non-relative cares for one or more children in their home—is a popular alternative [10,11]. In 2012, approximately 1,037,000 family homes in the United States provided such care [12]. FCCHs are unique from other types of care; they are generally classified as small businesses which are privately owned and operated by the provider primarily in their own home [12]. The majority of FCCH providers are female, between the ages of 30 and 60 years, and married or living with a partner [12]. The average household income of the majority of listed or registered FCCH providers was less than $45,000, and this is substantially lower for unlisted paid providers (less than $25,000), despite providing an average 40 to 54 h of care per week [12]. The National Survey of Early Care and Education (NSECE) found that only 34% of listed FCCH providers and 7% of unlisted paid FCCH providers have formal training in early childhood education [12]. There were only nine states that stipulated pre-service educational or training requirements and only 35 states required some clock hours of in-service training annually. Importantly, the content of the training is not stipulated and there are no states that require training specific to obesity prevention through healthy eating and regular physical activity.

To date, no studies have examined patterns of sedentary behavior in children attending FCCHs. A recent systematic review [10] of physical activity and sedentary behavior among preschool-aged children in FCCHs found only two studies [13,14] that reported children’s sedentary behavior. However, neither of these studies examined the frequency and duration of sedentary bouts, or assessed associations between the providers’ practices and child sedentary behavior. Therefore, the current study aimed to (1) describe the frequency and duration of sedentary bouts measured objectively by an accelerometer in a sample of children attending FCCHs; and (2) explore associations between selected child care practices and children’s sedentary behavior patterns.

## 2. Materials and Methods 

### 2.1. Participants and Settings

Registered FCCHs were recruited from five regional child care resource and referral (CCR&R) hubs serving seven economically diverse counties in Oregon, United States. FCCHs were randomly selected using the CCR&R database as a sampling frame. Before selection, the sample was stratified by the CCR&R hub and providers within each stratum were randomly sampled with a probability proportional to the total number of registered FCCHs operating in the hub. Initially, 63 FCCHs were enrolled in the study. Of those, five had too few children under their care when data collection began (<4 children) and two others closed their business before data collection, which left a sample of 56 FCCHs. All children between the ages of two and five years attending these FCCHs were invited to take part in the activity monitoring portion of the study. The number of eligible children within each FCCH ranged from one to six, with a median of four child participants per FCCH. The study was approved by the university’s institutional review board, and before participating, FCCH providers and the children’s parents provided written informed consent.

### 2.2. FCCH Practices Related to Movement Behaviors 

FCCH practices related to movement behaviors were assessed using the Nutrition and Physical Activity Self-Assessment for Child Care self-assessment instrument (NAPSACC-SA) [15]. The NAPSACC-SA instrument has been used in previous studies [11,16,17] and shown to be a reliable tool for measuring nutrition and physical activity (PA) policies and practices in child care settings. The NAPSACC-SA [15] contains 19 items related to movement behaviors: active play and inactive time (five items); electronic media use (three items); play environment (four items); supporting PA (two items); education for children and parents (four items); and PA policy (one item). To accommodate FCCH settings, the word “staff” was replaced by the word “I”. Responses to each item were recorded on four-point Likert-type scales, with the response of “1” indicative of marginally meeting child care standards, “2” indicative of meeting child care standards, “3” indicative of exceeding child care standards, and “4” indicative of far exceeding child care settings and using best practice. Following the approach used in previous studies [18,19], for each item, FCCH’s meeting or exceeding accepted child care standards were classified as “promoting PA” (PPA) or “not promoting PA” (non-PPA). 

### 2.3. Sedentary Bouts

Sedentary bouts were objectively measured using an ActiGraph GT1M accelerometer (ActiGraph Corporation, Pensacola, FL, USA), which has been shown to be a valid instrument for measuring PA and sedentary behavior in both toddlers and preschool-aged children [20,21]. Participating children wore the accelerometer for the duration of FCCH attendance across a randomly selected week. FCCH providers were provided an instruction sheet that explained how to attach and remove the accelerometer. At the beginning of each monitoring day, the provider attached the accelerometer to the child’s right hip via an adjustable elastic belt, noting the time of attachment, the identification number of the child, and the identification number of the accelerometer on the activity monitoring log. Accelerometers were not worn during nap times. Therefore, providers also recorded the start and end of daily nap time on the log. When the child departed, the child care provider removed the accelerometer and noted the time of departure on the log. 

Activity counts recorded in 15 s epochs were uploaded to a customized visual basic macro for determination of time in sedentary, light, and moderate-to-vigorous PA. Counts were classified into these categories using the cut-points developed by Pate and colleagues [20]. Non-wear time was quantified by summing the number of consecutive 0 counts accumulated in sequences of 10 min or longer. Children were included in the analyses if they had ≥3 days in which wear time was ≥75% of the attendance time [22]. Because children did not wear the accelerometers during nap time, these time periods were detected as non-wear and not classified as sedentary time. A sedentary bout was defined as a period of uninterrupted sedentary time with at least four consecutive 15 s epochs with less than 25 counts each epoch. The number of bouts and total sedentary time accumulated in bouts were calculated for bouts lasting 1.0–4.9 min (short), 5.0–9.9 min (medium), 10.0–14.9 min (long), and ≥15 min (extended).

### 2.4. Statistical Analyses

Mixed model analysis of variance (ANOVA) was used to compare the number of sedentary bouts and sedentary time accumulated in bouts in FCCHs classified as PPA and non-PPA FCCHs. Each model included the group (PPA vs. Non-PPA), child sex, and accelerometer wear time as fixed effects, and FCCH as a random effect to control for the clustering of children within FCCHs. For each comparison, Cohen’s d effect sizes were computed from the resultant F-statistic using the methods outlined by Cohen [23]. A final mixed model was analyzed in which FCCHs were categorized into two groups according to whether they were classified as PPA on ≥4 movement related practices that were statistically significant (*p* < 0.05) and had an effect size ≥0.30 in the initial analyses. All analyses were conducted in SAS (Version 9.3, SAS, Cary NC, USA) using PROC Mixed.

## 3. Results

Of the 56 FCCHs participating in the study, 53 completed the NAPSACC-SA and 41 completed both the NAPSACC-SA and the accelerometer protocol. Within these 41 FCCHs, a total of 187 children agreed to wear an accelerometer for the activity monitoring study. Of this number, 127 children (68 boys, 59 girls) with a mean (SD) age of 3.5 (± 1.1) years wore the accelerometer and had ≥3 valid monitoring days. Assessments were completed between March 2011 and April 2012. The majority of the FCCHs providers were non-Hispanic white (92%), aged ≥40 years (53%), and had attained a high school diploma or equivalent. Detailed characteristics of all participating FCCHs and providers are presented in Table 1.

On average, the total time spent in sedentary, light-intensity, and moderate-to-vigorous physical activity was 181 ± 64, 127 ± 38, and 49 ± 22 min per day, respectively. Table 2 reports the number of sedentary bouts and total sedentary time accumulated in short, medium, long, and extended bouts, by child gender. Overall, 84% and 87% of the sedentary bouts in boys and girls, respectively, were short bouts of less than five minutes. Compared with boys, girls had significantly more sedentary bouts (41.6 vs. 36.6; *p* = 0.002), particularly short bouts of less than five minutes (36.0 vs. 30.8; *p* < 0.001). Total sedentary time accumulated in short bouts was significantly greater in girls compared with boys (*p* < 0.001); however, sedentary time accumulated in medium and long bouts was comparable between boys and girls. Sedentary time accumulated in extended bouts was greater in boys than in girls; however, this difference was not statistically significant.

Table 3 shows the number of sedentary bouts and total sedentary time accumulated in short and medium bouts in FCCHs classified as PPA versus non-PPA. Significant differences in child sedentary behavior were observed for 9 of the 14 movement-related child care practices. Children had significantly fewer sedentary bouts, and/or significantly less sedentary time accumulated in short and medium bouts in FCCHs where outdoor active play was provided to all children on a daily basis, a variety of fixed and portable play equipment was available, indoor play space was available for when the weather was not suitable to go outdoors, children were consistently not seated for more than 30 min at a time, computer use was limited to only a few times a week, the provider routinely played with children during active free play time, the provider read books or played games with PA or exercise themes, and/or education about PA was offered to parents. For these practices, Cohen’s d effect sizes ranged from 0.38 (provision of portable play equipment and medium sedentary bouts) to 0.79 (limiting computer use for educational purposes or games and short sedentary bouts). For the child care practices with statistically significant differences for the number of sedentary bouts and/or sedentary time accumulated in short or medium bouts, the average (95% confidence interval (CI)) effect size was 0.53 (0.49–0.58). Cohen [23] suggests that effect sizes of 0.20 are small, 0.50 are medium, and 0.80 are large. Thus, the observed differences in sedentary bouts between FCCHs classified as PPA and non-PPA were, on average, medium in magnitude and meaningful. Children attending FCCHs classified as PPA on four or more of these practices exhibited approximately 12 fewer sedentary bouts per day, and accumulated approximately 30 min less of sedentary time per day in short and medium bouts. An additional table reporting F-statistics, numerator, and denominator degrees of freedom, *p*-values, and Cohen’s d for each child care practice is provided in the Supplementary Table S1.

## 4. Discussion

Public health officials recommend young children should not be sedentary for more than one hour at a time [4]. This study described the frequency and duration of sedentary bouts in children attending FCCHs in the northwestern region of the United States. Consistent with the results of studies conducted in center-based child care settings [24], children attending FCCHs accumulated very little sedentary time in long or extended bouts lasting more than 10 min. Children in the current study mostly accumulated sedentary time in short bouts (1–4 min). These findings are consistent with the results of previous studies documenting the sporadic and pulsatile nature of young children’s movement behaviors [25]. 

Significantly fewer sedentary bouts were recorded among children attending FCCHs where providers often or always played with children during active free play. This finding highlights the positive influence of adult co-participation on children’s movement behaviors, and further endorses the need to increase support for FCCH providers to engage in active play with the children under their care. However, a previous study reported that the majority of FCCHs failed to meet established child care standards for structured play, screen time, and indoor play space [26]. Additionally, a focus group study involving 32 FCCH providers identified lack of appropriate curricular ideas, limited funds, little physical play space, and minimal parental support as significant barriers to the promotion of regular PA in FCCHs [27]. A previous intervention study, where FCCHs were supported by child care trainers through a process of self-evaluation, goal setting, action plan development, and progress evaluation, was shown to be effective in improving healthy eating and PA scores in children [11]. Therefore, professional development programs to address the barriers and assist FCCHs in adopting and implementing these key practices may help to reduce child sedentary time while in care. Future intervention studies targeting family day care settings should test this hypothesis.

Children attending FCCHs that met or exceeded child care standards for outdoor active play, active play using portable play equipment, offering a variety of fixed play equipment, and/or adequate indoor play space had significantly fewer sedentary bouts. Research conducted on center-based care and FCCHs shows having an adequate play space and using age-appropriate equipment can enhance children’s participation in moderate-to-vigorous physical activity [28,29]. These findings suggest the availability of a supportive environment for PA and the utilization of play equipment may help prevent prolonged bouts of sedentary behavior in children attending FCCHs. While interventions to increase PA-related knowledge and skills of FCCH providers are needed, it is also recommended that future intervention studies help providers create home care environments that facilitate PA and deter sedentary behaviors.

Our findings are consistent with those reported by Kuzik and colleagues [24], who objectively measured bouts of sedentary behavior in a sample of toddlers and preschoolers attending center-based child care in Alberta, Canada. In that study, sedentary behaviors over the child care day were accumulated primarily in short bouts, lasting 1 to 4 min, with almost no engagement in extended bouts lasting 15 min or longer. On the basis of a standard 5.5 h child care day, Canadian children accumulated a total of 37 bouts of sedentary behavior lasting 1 to 4 min and a total of 5 bouts lasting 6 to 9 min [24]. In comparison, children participating in the current study accumulated, on average, 33 sedentary bouts lasting 1 to 4 min, and 3 bouts lasting 5 to 9 min. Notably, in both studies, the average number of sedentary bouts lasting 10 min or more was just over two per day. While the results of the two studies were remarkably similar, there was, however, an important difference in relation to gender differences in sedentary behavior patterns. Unlike the current study, which found boys to accumulate significantly fewer sedentary bouts lasting 1 to 4 min, and significantly less sedentary time accumulated in bouts than girls, the Kuzik study observed the frequency of sedentary bouts to be similar among boys and girls. This discrepancy may be a result of differences in the minimal accelerometer wear time requirement (≥75% attendance time on ≥3 days vs. ≥1 h of wear time on ≥3 days), differences in accelerometer model (ActiGraph vs. Actical) and their associated cut-points for delineating light intensity PA from sedentary behavior, or true population differences in gender-specific patterns of sedentary behavior among young children.

The current study contributes to the evidence base on sedentary behavior patterns among preschoolers, which remains an under-studied area [24,30]. Strengths include the objective measurement of sedentary bouts using an accelerometer, which is a validated measure of movement behavior in young children [20,31,32], and the evaluation of sedentary time accumulated in bouts of various durations. Offsetting these strengths were a number of limitations. First, hip mounted accelerometers cannot detect posture, and hence cannot reliably distinguish between quiet sitting and motionless standing. Therefore, the estimates of sedentary time should be viewed with some caution. Second, although the sedentary bouts were measured objectively, accelerometers cannot determine the types of sedentary behaviors in which children were engaged. It should be acknowledged that some sedentary behaviors have an educative value, and accelerometer data does not distinguish sedentary time that is educational, such as reading stories, drawing, or solving puzzles, from time that is non-educational, for example, watching television for leisure or streaming cartoons on a tablet or smartphone. Third, data for this study were collected between 2011 and 2012. However, because the policy and practice environment for FCCHs has changed little since this time, the results are informative and remain relevant to contemporary FCCH providers seeking to promote healthy movement behaviors in the young children in their care. Nevertheless, additional studies involving contemporary FCCH samples are needed to confirm our findings. Fourth and finally, child care practices related to PA and sedentary behaviors were self-reported by the FCCH provider, and thus are subject to self-reporting bias. However, the risk of bias is deemed low given that the NAPSACC-SA instrument has been validated and shown to be a reliable tool for measuring PA practices in child care settings [11,16,17].

## 5. Conclusions

Children in FCCHs accumulate sedentary time in short bouts lasting 4 min or less, with very little sedentary time accrued in bouts lasting more than 10 min. Children exhibited fewer sedentary bouts and accumulated less sedentary time in bouts in FCCHs in which providers joined in during active play time, offered supportive PA environments (adequate fixed and portable play equipment, appropriate indoor space for PA), limited children sitting time to less than 30 min at a time, or offered PA education to parents. Programs to reduce sedentary time while in care should support FCCHs in implementing these practices. 

## Figures and Tables

**Table 1:** Characteristics of family child care homes (FCCHs) and providers. NAPSACC-SA, Nutrition and Physical Activity Self-Assessment for Child Care self-assessment instrument.

Characteristics of Family Child Care Homes	FCCHs Completed NAPSACC-SA (n = 53)	FCCHs Completed NAPSACC-SA and Accelerometer Protocol (n = 41)
Years of operation, median (IQR)	10 (5–14)	10 (5–15)
Number of children aged 2–5 years under provider’s care, median (IQR)	4 (3–5)	3 (2–4)
Provider age (years), n (%)		
Under 30	3 (6)	1 (2)
30–34	8 (15)	6 (15)
35–39	15 (28)	12 (29)
40 or above	27 (51)	22 (54)
Highest education level of provider, n (%)		
High school diploma or GED	35 (66)	28 (68)
College or associate degree	10 (19)	7 (17)
University degree (Bachelor)	8 (15)	6 (15)
Non-Hispanic white, n (%)	49 (93)	39 (95)

IQR: interquartile range (difference between quartile 3 and quartile 1); GED: general educational development; NAPSACC-SA: Nutrition and Physical Activity Self-Assessment for Child Care self-assessment instrument.

**Table 2:** Daily number of sedentary bouts and sedentary time in bouts by child gender.

	Total (n = 127)	Boys (n = 68)	Girls (n = 59)
Number of sedentary bouts per day, mean (95% CI)			
All sedentary bouts (total) *	38.9 (36.6–41.2)	36.6 (34.4–38.7)	41.6 (39.3–43.9)
Short sedentary bouts (1–4 min) *	33.2 (31.2–35.3)	30.8 (28.8–32.8)	36.0 (33.9–38.2)
Medium sedentary bouts (5–9 min)	3.3 (3.0–3.7)	3.3 (2.8–3.7)	3.4 (3.0–3.9)
Long sedentary bouts (10–14 min)	1.0 (0.9–1.1)	1.0 (0.9–1.2)	0.9 (0.8–1.1)
Extended sedentary bouts (≥15 min)	1.3 (1.1–1.5)	1.4 (1.2–1.6)	1.2 (1.0–1.4)
Total daily sedentary time (minutes) accumulated in bouts, mean (95% CI)			
Short sedentary bouts (1–4 min) *	61.8 (58.8–64.8)	57.2 (53.3–61.1)	67.1 (62.9–71.3)
Medium sedentary bouts (5–9 min)	25.4 (21.4–29.4)	25.1 (20.6–29.6)	25.7 (21.1–30.3)
Long sedentary bouts (10–14 min)	14.2 (11.7–16.8)	14.8 (12.0–17.6)	13.7 (10.9–16.5)
Extended sedentary bouts (≥15 min)	38.0 (33.1–42.9)	41.0 (34.3–47.7)	34.6 (27.4–41.7)

* *p* < 0.05 between gender. CI: confidence interval.

**Table 3:** Comparison of number of sedentary bouts and sedentary time in short and medium bouts in FCCHs classified as PPA vs. non-PPA.

Physical Activity Practice	Number of Sedentary Bouts (n)				Sedentary Time in Short Bouts (min)				Sedentary Time in Medium Bouts (min)			
	PPA	NPPA	*p*	d	PPA	NPPA	*p*	d	PPA	NPPA	*p*	d
Structured PA (adult-led) is provided for all children daily	39.1 ± 1.0	38.5 ± 1.3	0.725	0.06	62.2 ± 1.8	61.1 ± 2.4	0.716	0.07	25.1 ± 2.2	26.2 ± 2.6	0.609	0.09
Outdoor active play is provided for all children daily	38.3 ± 1.2	43.9 ± 1.7	0.004	0.64	60.0 ± 1.6	68.7 ± 3.1	0.016	0.54	25.0 ± 2.1	27.3 ± 3.0	0.385	0.19
Children are seated (excluding nap time) more than 30 min at a time once per week or less	38.6 ± 1.4	43.2 ± 1.8	0.006	0.52	59.9 ± 2.1	67.2 ± 2.4	0.017	0.45	24.2 ± 2.1	31.2 ± 2.7	0.002	0.59
Children are allowed to watch TV and videos or play video games less than four times per week	38.8 ± 1.6	40.0 ± 1.5	0.505	0.12	60.2 ± 2.5	62.7 ± 1.8	0.413	0.15	25.5 ± 2.3	25.1 ± 2.4	0.867	0.03
Children may use a computer for educational purposes or games less than four times per week	37.5 ± 0.9	44.0 ± 1.7	0.001	0.74	59.2 ± 1.6	71.6 ± 3.0	<0.001	0.79	25.7 ± 2.1	24.5 ± 2.9	0.653	0.10
Fixed play equipment (swings, slides, overhead ladders) is suitable and available	39.0 ± 1.4	43.3 ± 2.0	0.017	0.48	60.4 ± 1.7	65.8 ± 2.8	0.104	0.33	25.0 ± 2.1	28.9 ± 2.9	0.116	0.31
Active play using portable play equipment is provided daily	38.2 ± 1.3	42.4 ± 1.7	0.012	0.47	59.4 ± 1.8	66.2 ± 2.4	0.024	0.42	24.3 ± 2.1	28.7 ± 2.6	0.044	0.38
Indoor play space is available and suitable for all activities	37.1 ± 1.7	39.5 ± 1.0	0.215	0.24	58.6 ± 3.0	62.8 ± 1.7	0.230	0.25	19.7 ± 2.8	26.0 ± 1.9	0.015	0.52
Provider often or always plays with children during active (free) play time	37.4 ± 1.3	42.7 ± 1.5	0.001	0.46	57.8 ± 1.8	67.7 ± 2.2	0.001	0.62	23.4 ± 2.2	26.0 ± 1.7	0.007	0.50
Provider receives training or attend workshops on PA at least once a year	37.9 ± 1.8	40.1 ± 1.3	0.245	0.24	58.7 ± 2.9	62.9 ± 1.7	0.216	0.25	23.8 ± 2.6	26.5 ± 2.3	0.278	0.22
Provider reads books and plays games with PA or exercise themes	39.6 ± 1.5	44.0 ± 2.3	0.023	0.46	62.0 ± 2.6	71.4 ± 4.0	0.006	0.56	25.4 ± 2.1	26.9 ± 3.2	0.581	0.11
Education about PA is offered to parents through flyers, handouts, and newsletters	35.5 ± 1.6	40.4 ± 1.1	0.007	0.55	56.4 ± 2.8	63.7 ± 1.7	0.027	0.45	21.6 ± 2.7	26.7 ± 2.1	0.038	0.42
Provider has a comprehensive written policy on PA	39.2 ± 1.8	39.0 ± 1.1	0.908	0.03	61.8 ± 3.3	61.8 ± 1.6	0.991	0.00	23.7 ± 2.7	27.1 ± 2.4	0.231	0.26
Four or more significant PPA characteristics	37.5 ± 0.8	49.6 ± 2.1	<0.001	1.50	59.3 ± 1.4	80.3 ± 3.9	<0.001	1.41	21.1 ± 1.1	33.5 ± 3.0	<0.001	1.10

FCCH: family child care home; n: number; PA: physical activity; PPA: promoting physical activity; NPPA: not promoting physical activity; *P*: *p*-value; d: Cohen’s d; TV: television. Results are presented as mean and standard errors are adjusted for sex, wear time, and clustering of child within the FCCH, except for the number of FCCHs, which is presented as number (percentage). Short and medium sedentary bouts are expressed as minutes of sedentary time. All F-tests for PPA vs. NPPA were conducted with 1 df numerator and 122 df denominator = F _(1,122)_.

## Supplementary Materials

The following are available online at https://www.mdpi.com/1660-4601/17/2/549/s1, Table S1: Detailed statistical results for mixed model ANOVAs comparing PPA vs. Non-PPA FCCHs.
