# Supplementary material for: Patterns and Correlates of Sedentary Behavior in Children Attending Family Child Care
Source: Int J Environ Res Public Health. 2020 Jan 15;17(2):549. doi: 10.3390/ijerph17020549 (PMC7014021; doi:10.3390/ijerph17020549)
Supplement: Supplementary file 1 [file ijerph-17-00549-s001.pdf]

**Table 1.** Detailed statistical results for mixed model ANOVAs comparing PPA vs Non-PPA FCCHs.

| Physical activity practice | # sedentary bouts (n) | | | | SED time in short bouts (min) | | | | SED time in medium bouts (min) | | | |
|---|---|---|---|---|---|---|---|---|---|---|---|---|
| | F-Stat | df | *P* | d | F-Stat | df | *P* | d | F-Stat | df | *P* | d |
| Structured PA (adult-led) is provided for all children daily | 0.12 | 1,122 | 0.725 | 0.06 | 0.13 | 1,122 | 0.716 | 0.07 | 0.26 | 1,122 | 0.609 | 0.09 |
| Outdoor active play is provided for all children daily | 8.46 | 1,122 | 0.004 | 0.64 | 6.01 | 1,122 | 0.016 | 0.54 | 0.76 | 1,122 | 0.385 | 0.19 |
| Children are seated (excluding nap time) more than 30 minutes at a time once per week or less | 7.74 | 1,122 | 0.006 | 0.52 | 5.83 | 1,122 | 0.017 | 0.45 | 9.81 | 1,122 | 0.002 | 0.59 |
| Children are allowed to watch TV, videos or play video games less than 4 times per week | 0.45 | 1,122 | 0.505 | 0.12 | 0.67 | 1,122 | 0.413 | 0.15 | 0.03 | 1,122 | 0.867 | 0.03 |
| Children may use a computer for educational purposes or games less than 4 times per week | 11.52 | 1,122 | <0.001 | 0.74 | 13.3 | 1,122 | <0.001 | 0.79 | 0.20 | 1,122 | 0.653 | 0.10 |
| Fixed play equipment (swings, slides, overhead ladders) is suitable and available | 5.82 | 1,122 | 0.017 | 0.48 | 2.69 | 1,122 | 0.104 | 0.33 | 2.51 | 1,122 | 0.116 | 0.31 |
| Active play using portable play equipment is provided daily | 6.54 | 1,122 | 0.012 | 0.47 | 5.25 | 1,122 | 0.024 | 0.42 | 4.11 | 1,122 | 0.044 | 0.38 |
| Indoor play space is available and suitable for all activities | 1.56 | 1,122 | 0.215 | 0.26 | 1.46 | 1,122 | 0.230 | 0.25 | 6.06 | 1,122 | 0.015 | 0.52 |
| Provider often or always plays with children during active (free) play time | 10.66 | 1,122 | 0.001 | 0.59 | 11.72 | 1,122 | 0.001 | 0.62 | 7.48 | 1,122 | 0.007 | 0.50 |
| Provider receives training or attend workshops on PA at least once a year | 1.37 | 1,122 | 0.245 | 0.24 | 1.55 | 1,122 | 0.216 | 0.25 | 1.19 | 1,122 | 0.278 | 0.22 |
| Provider reads books and plays games with PA or exercise themes | 5.30 | 1,122 | 0.023 | 0.46 | 7.69 | 1,122 | 0.006 | 0.56 | 0.31 | 1,122 | 0.581 | 0.11 |
| Education about PA is offered to parents through flyers, handouts, newsletters | 7.42 | 1,122 | 0.007 | 0.55 | 5.01 | 1,122 | 0.027 | 0.45 | 4.42 | 1,122 | 0.038 | 0.42 |
| Provider has a comprehensive written policy on PA | 0.01 | 1,122 | 0.908 | 0.03 | 0.00 | 1,122 | 0.991 | 0.00 | 1.45 | 1,122 | 0.231 | 0.26 |
| Four or more significant PPA characteristics | 29.53 | 1,122 | <0.001 | 1.50 | 26.25 | 1,122 | <0.001 | 1.41 | 16.80 | 1,122 | <0.001 | 1.10 |
